# The “inherent vice” in the anti-angiogenic theory may cause the highly metastatic cancer to spread more aggressively

**DOI:** 10.1038/s41598-017-02534-1

**Published:** 2017-05-24

**Authors:** Denian Wang, Chun Tan, Fei Xiao, Lan Zou, Lijun Wang, Yong’gang Wei, Hanshuo Yang, Wei Zhang

**Affiliations:** 1 Molecular Medicine Research Center, State Key Laboratory of Biotherapy, West China Hospital, Sichuan University and Collaborative Innovation Center, Chengdu, 610041 Sichuan P.R. China; 2 Department of Intensive Care Unit of Gynecology and Obstetrics, West China Second University Hospital, Sichuan University, Chengdu, 610041 Sichuan P.R. China; 3 Department of Mathematics, Sichuan University, Chengdu, Sichuan 610064 P.R. China; 4 Department of Liver Surgery, West China Hospital, Sichuan University, Chengdu, 610041 Sichuan P.R. China; 5 State Key Laboratory of Biotherapy and Cancer Center, West China Hospital, Sichuan University and Collaborative Innovation Center, Chengdu, Sichuan 610064 P.R. China

## Abstract

Although anti-angiogenic (AA) therapy is widely used in clinical practice, it is often challenged by insufficient efficacy and intrinsic resistance. Some studies have reported that AA therapy can even increase tumor metastasis. However, whether this is due to a specific AA drug causing a specific tumor to metastasize or because the anti-angiogenic theory has some “inherent vice” that may inevitably lead to tumor dissemination remains a mystery. Herein, we designed a model that completely blocks tumor blood supply using a physical barrier to examine tumor behavior in such circumstances. Surprisingly, we found that cutting off the blood supply could neither eliminate the primary tumor cells nor prevent local invasion or formation of distant metastases. By using a mathematical method to simulate tumor behavior, we found that blocking tumor blood supply may lead to an inevitable consequence: the cells that can tolerate blood deficiency are “naturally selected” and survive, whereas a portion of cells are promoted to escape from the “starvation” area by the consistent environmental stress until they are spread throughout the body. This may be an intrinsic disadvantage of the AA strategy, which will inevitably cause the tumor, particularly highly metastatic tumors, to spread more aggressively.

## Introduction

Like normal cells, cancer cells need nutrients and oxygen and the ability to evacuate metabolic wastes to support their robust growth. The neo-vasculature generated by the process of angiogenesis addresses those needs^1, 2^. Efforts in targeting angiogenesis to inhibit tumor growth by reducing its blood supply have thereby attracted great interest. Many patients with cancer have benefited from anti-angiogenic (AA) therapies since 2004, when bevacizumab (a monoclonal-antibody against vascular endothelial growth factor (VEGF)) was approved by the U.S. FDA. Unfortunately, this strategy is now challenged by insufficient efficacy and resistance^3^. These AA agents, whether used as a mono-therapy or combined with chemotherapy, only provide limited survival benefits, on the order of weeks to months, or, in some cancers, show no efficacy at all^4^. The reduction of the primary tumor mass after AA therapy was confirmed in most preclinical studies and clinical trials. However, the primary tumor reduction is usually followed by tumor relapse^5, 6^ with increased tumor invasiveness and metastasis^7, 8^. The response of the primary tumor to AA therapy does not consistently correlate with improved survival, and survival is worsened in certain settings^9^.

There are some possible mechanisms to explain the lower than expected efficacy of AA therapy, such as revascularization by alternative pro-angiogenic signals, protection of tumor vasculature by increased pericyte coverage, and intrinsic unresponsiveness^10^. However, these theories were primarily focused on the idea that the current AA therapies were not potent enough to prevent the tumor from getting an adequate blood supply. This leads one to think: is it possible to create an environment in which the tumor blood supply is completely cut off? If it is possible, will it eliminate the primary tumor and prevent the tumor from metastasizing?

To date, more than 40 molecules have been determined to play a role in angiogenesis^4, 11^. Unless an AA agent can target all of them, it is nearly impossible to completely block tumor angiogenesis. To circumvent this obstacle, we designed a model in which the blood flow from the host to the tumor was completely blocked using a physical barrier instead of AA drugs. On the theoretical basis of the anti-angiogenic strategy, AA therapy does not forbid fluid/molecule exchange at the junction of the tumor and host tissues. Thus, a physical barrier used to disconnect the tumor and host vasculature should not be impenetrable, but should have micro-channels that allow the exchange of materials. This model therefore has the following features: it can completely block blood flow from host to tumor, but it does not affect the host vasculature, and it does not forbid substance exchange at the tumor/host border. Those characteristics fit the expectation of ideal AA therapy. With the aid of this model, we might be able to learn about what would occur if the tumor blood supply is completely cut off.

On the other hand, from a theoretical standpoint, we realized that even if a potent AA therapy that can totally block the tumor blood supply exists, it cannot prevent the tumor cells from migrating because this process is independent of angiogenesis. In addition, under environmental stress, tumor cells that cannot tolerate the blood deficiency may escape from the “starvation” area to the adjacent, well-perfused host tissues, and they may invade the host vasculature via their own motility. AA agents are designed to target pro-angiogenic molecules, which are mainly expressed in some tumor cells themselves, endothelial cells, and some stromal cells. Unless the pro-angiogenic molecules are also expressed in the tumor cells and involved in tumor cell movement, AA agents cannot prevent tumor cell migration and invasion. Although some AA agents may transiently improve the cytotoxic drug delivery via “vessel normalization”, the potent and high-dose of AA agents is supposed to cause severe hypoxia and nutrient deficiency within the tumor. If the environmental stress forces the cancer cells to escape from the original zone, and AA agents cannot prevent the cancer cell migration, then eventually, the cancer cells will spread throughout the body. In other words, the more potent the AA agents are, the more likely it is that the cancer cells are forced to escape and disseminate. This may be an intrinsic disadvantage in the anti-angiogenic theory, which may make it impossible to prevent, and might even promote, tumor invasion and metastasis.

Previous studies have shown that some potent AA therapies increased the tumor local invasion and accelerated distant tumor metastasis in mouse models^7, 8^. However, whether this is due to a certain AA agent causing a certain type of tumor to metastasize or due to the anti-angiogenic theory having its own “inherent vice” remains undetermined. If the anti-angiogenic strategy has a theoretical flaw, even if we could develop a “perfect” AA therapy that could completely cut off the tumor blood supply without attacking normal blood vessels, then, eventually, it would promote tumor invasion and metastasis. In this study, we sought to establish a novel model to mimic an environment in which the tumor blood supply is completely blocked and examine the behavior of tumor cells in this blood deficient environment. Our purpose is to use the experimental data to explore whether the anti-angiogenic strategy has a theoretical defect and determine if it causes the failure of AA therapy in the treatment of cancer.

## Results

### Blocking the blood supply of solid tumors using a physical barrier

**Figure 1:**
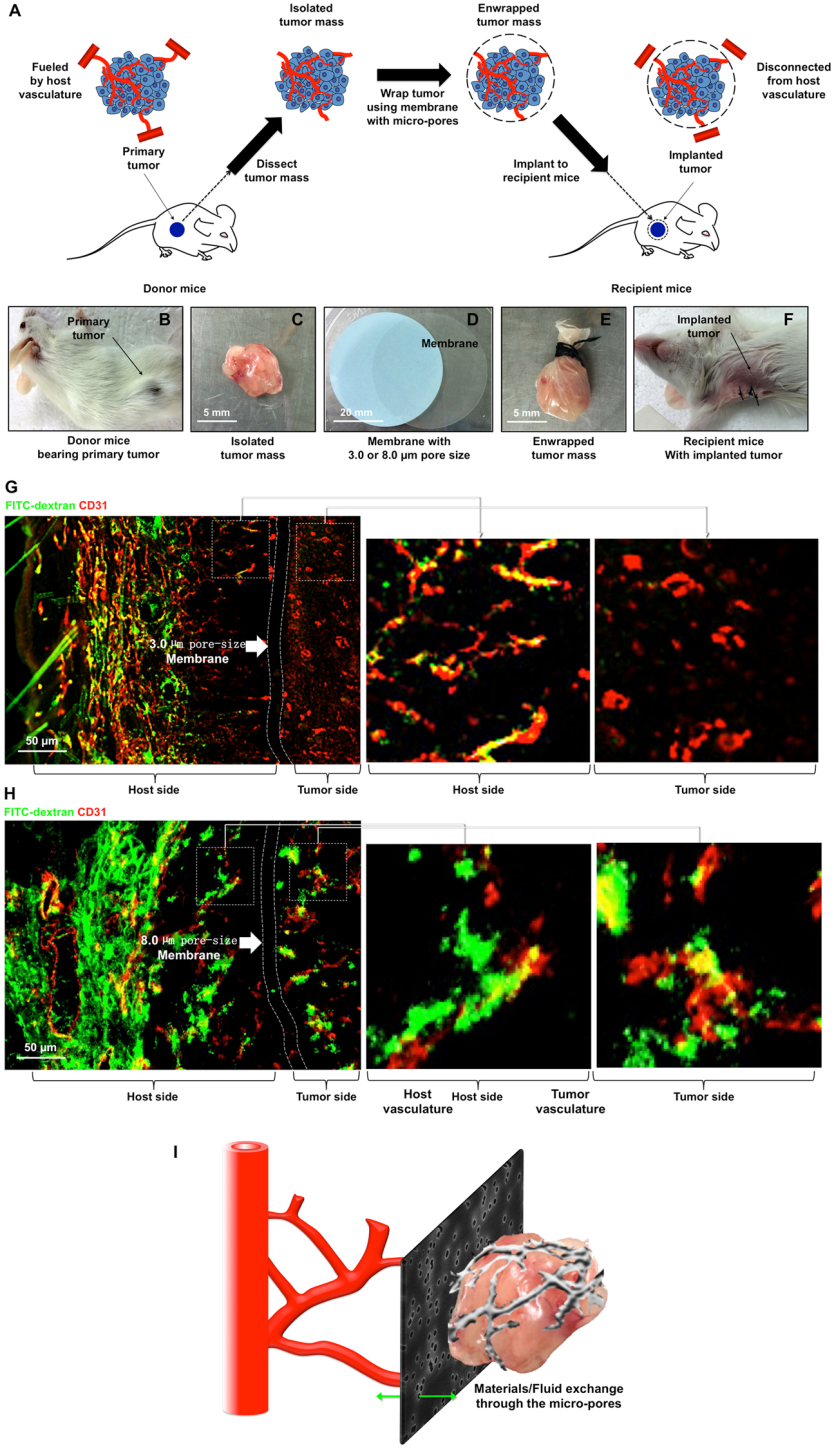
Cutting off tumor blood supply by disconnecting the vasculature between tumor and host using a physical barrier. (**A**) The schematic shows the experimental procedure. To establish the primary tumor, 2.5 × 10^6^ 4T1 cells were injected into a Balb/c mouse (**B**). When the tumor reached a diameter of approximately 5 mm, it was surgically removed **(C)** and wrapped with a hydrophilic polycarbonate membrane with 3.0 μm micro-pores (upper panel: electron microscopy image of the surface of the membrane) **(D)**. The membrane-wrapped tumor mass (**E**) was then implanted into another healthy recipient mouse **(F)**. (**G,H**) Three weeks after tumor implantation, FITC-conjugated dextran (2000 kD, green) was injected into the mice through the left ventricle and allowed to circulate for 10 min. The 3.0 μm (**G**) or 8.0 μm (**H**) membrane-wrapped tumors were then removed completely, including the adjacent tissues, and stained with CD31 (red) to visualize the entire vasculature. As shown in the right panels, the tissues in the host side were well perfused, but green/red co-localization was not observed on the tumor side of the barrier. (**I**) Under this condition, the tumor and host vasculature was completely disconnected by the physical barrier; on the other hand, the molecules and fluids can cross the border between the host and tumor through the micro-pores.

We chose 4T1 mouse breast cancer to establish the tumor model because it produces a highly metastatic solid tumor that can spontaneously metastasize to the lung, which closely mimics highly metastatic human breast cancer^12, 13^. The donor mice were injected subcutaneously with 4T1 cells to establish the primary tumor. When the tumor reached a diameter of approximately 5 mm, it was surgically removed and carefully wrapped with the polycarbonate membrane with a pore size of 8.0 μm or 3.0 μm (Supplementary Fig. S1). Each wrapped tumor was implanted into another healthy mouse recipient, as shown in Fig. 1A–F. In theory, the capillaries (4~8 μm) generated by angiogenesis were unable to cross the membrane through the micro-pores less than 3.0 μm to connect the host and tumor vasculature (Fig. 1I). To test this, FITC-dextran was injected intravenously into the recipient mice to mark the perfused vessels. As shown in Fig. 1G and H, the FITC was not observed in the tumors encapsulated with 3.0 μm membrane (but not 8.0 μm), indicating that the blood flow from host to tumor was cut off. On the other hand, the 3.0 μm pores allowed the exchange of molecules and fluids at the border of host and tumor. Thus, this “physical cage” simulates an environment in which blood flow from host to tumor is completely blocked, but the local substance/fluid exchange at the tumor/host border is not forbidden. Here we didn’t use the impenetrable material to encapsulate the tumor, because it would be equivalent to completely isolating the tumor from the host, which is no different than a surgical resection, and definitely the tumor cannot survive.

### Cutting off tumor blood supply was unable to eliminate the primary tumor cells

**Figure 2:**
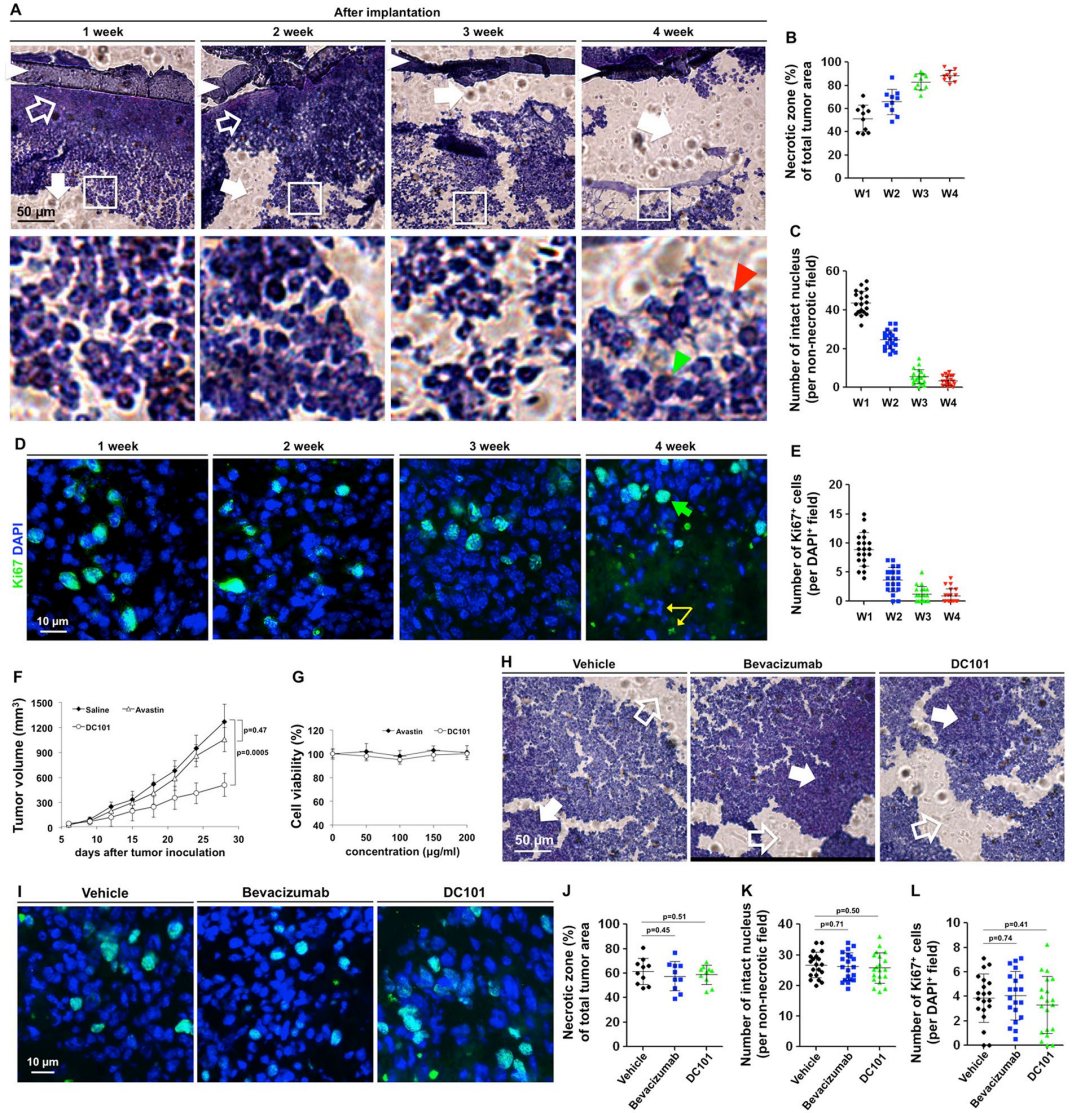
The tumor cells were not eliminated, even when their blood supply was entirely blocked. (**A**) The tumor samples were collected 1~4 weeks after implantation and stained with H&E. The open arrows indicate the coagulative necrosis, and the solid arrows indicate the liquefactive necrosis (shown as empty spaces, which were caused by the sectioning process). The white arrowheads indicate the membrane-barrier, the red arrowhead indicates the cell debris, and the green arrowhead indicates the cells with an intact nucleus. (**B**) The mean necrotic area of the samples was quantified using 10 slides (2 slides from each sample) per group. (**C**) The number of intact nuclei per non-necrotic field was counted using 20 randomly chosen fields (4 fields from each sample) per group. (**D**) The tumor samples were stained with Ki67 and DAPI. Note that the yellow arrows indicate the un-overlapped green/blue fluorescence, which were not considered active cells but only cell debris. (**E**) The number of Ki67^+^ cells (with an intact nucleus) per DAPI^+^ field was quantified in 20 randomly chosen fields. (**F**) Bevacizumab (10 mg/kg), DC101 (40 mg/kg), or vehicle (saline) was directly injected into the tumors with blood supply (the orthotopic tumor) or without blood supply (the encapsulated tumor). The tumor volume was measured every 3 days. (**G**) 4T1 cell viability was measured after the treatment of bevacizumab and DC101. (**H**–**L**) The encapsulated tumors were collected 2 weeks after drug injection and stained with H&E (**H**), or Ki67 (**I**). The mean necrotic area (**J**), the number of intact nuclei per non-necrotic field (**K**), and Ki67^+^ cells (**L**) of the samples was quantified as described above. The data are presented as scatter plots with means ± SD.

We then examined whether the tumor would be destroyed under these conditions. H&E staining showed that the necrotic area within the tumor increased over time, and the number of cells with an intact nucleus decreased gradually after the tumor blood supply was blocked (Fig. 2A–C). However, it did not surprise us that the number of live tumor cells decreased over time. What particularly interested us was that some of the tumor cells could still be active even though their blood supply was blocked for 4 weeks. The Ki67 staining showed that there were still some cells, although only a few, that had the ability to proliferate after being deprived of a blood supply for 4 weeks (Fig. 2D,E). In addition, some Ki67^+^ cells resided deep in the core of the encapsulated tumor, which were unlikely to be fused by the external vessels outside the barrier (Supplementary Fig. S2). These results suggest that even completely blocking the blood supply may not be sufficient to eliminate the primary tumor.

Our model is using physical barrier to block the blood flow. We then sought to investigate whether AA agents affects tumor growth with or without blood supply in addition to pruning vasculature. Two AA agents, bevacizumab (humanized mAb against human-VEGF) and DC101 (mAb against mouse-VEGFR2), were used in this experiment. The encapsulated tumors were implanted into the recipient mice, and the naturally growing tumors (with blood supply) were used as the control. The AA agents were directly injected into the tumors. Herein, we didn’t use the intravenous injection because the physical barrier will block the blood flow from the host, thus the drugs could not be delivered into the encapsulated tumors through the blood vessel system of the host. We found that bevacizumab barely affected the growth of the control tumor (with blood supply), which may be due to its weak interaction ability to mouse-VEGF^14^. On the other hand, DC101 significantly inhibited the control tumor growth (Fig. 2F). However, this effect may contribute to the inhibition of angiogenesis, because both Avastin and DC101 had no direct killing activity against the 4T1 cells (Fig. 2G). In addition, we examined the necrotic area, the number of cells with intact nucleus, the Ki67-positive cells, and the apoptotic area in the encapsulated tumors, and found no significant difference between the vehicle-, Avastin-, or DC101-treated tumors (Fig. 2H–L and Supplementary Fig. S3). The results suggested that the AA agents might no direct affect the tumor growth.

### Cutting off blood supply did not prevent local invasion or distant metastasis

**Figure 3:**
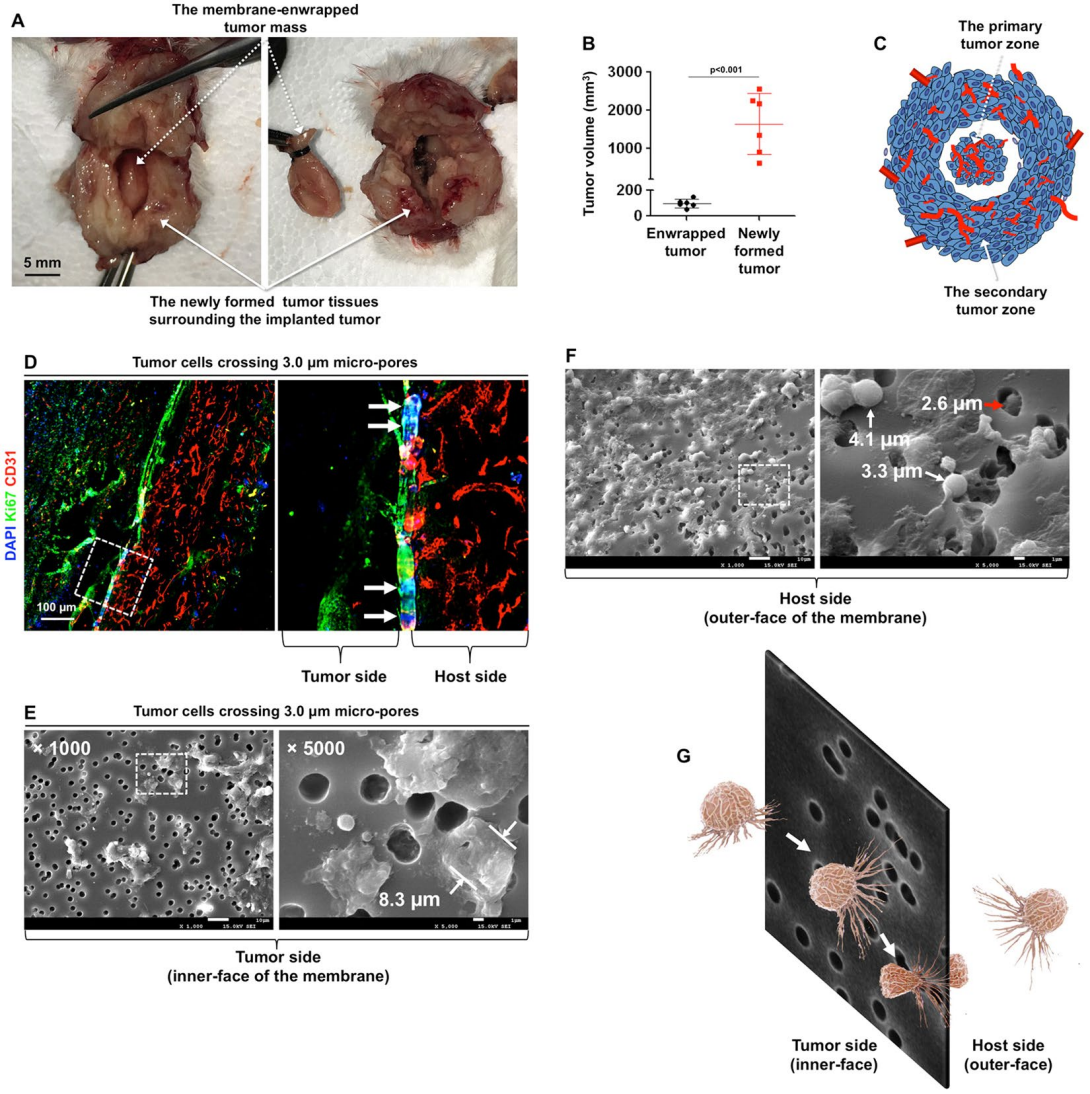
Cutting off tumor blood supply could not prevent local invasion of tumor. (**A**) The membrane-wrapped tumor was surrounded by newly formed tumor tissue 4 weeks after implantation. The dashed arrow indicates the implanted tumor. The solid arrow indicates the newly formed tumor tissue. (**B**) The volume of the original implanted-tumors and the adjacent tumor tissue was quantified using 6 mice. (**C**) The schematic shows that the newly formed tumor tissue surrounded the implanted primary tumor. (**D**) Three weeks after tumor implantation, the implanted tumors and the adjacent tissue were isolated and stained with CD31 (red), Ki67 (green), and DAPI (blue). The arrows indicate the cell nuclei crossing through the micro-pores of the membrane. (**E**,**F**) Electron microscopy shows the inner-face (tumor side) and outer-face (host side) of the 3.0 μm membrane. Red arrows indicate the cells in the micro-pores. (**G**) The diagram shows that tumor cells cross the micro-pores via morphological changes.

We planned to prolong the observation time, but found that the recipient mice started to die at week 5. A necropsy revealed that the membrane-wrapped primary tumor was surrounded by newly formed tumor tissue (Fig. 3A and Supplementary Fig. S4) with a volume much larger than that of the primary tumor (Fig. 3B,C). To determine whether the primary tumor cells were able to cross the physical barrier, we examined the border between the host and tumor 3 weeks after implantation. Fluorescence microscopy showed a number of tumor cells travelling through the 3.0 μm micro-pores on the membrane (Fig. 3D). Using electron microscopy, we found that cells on the tumor side are larger than cells on the host side (Fig. 3E,F), and the cells entering the micro-pores fit the 3.0 μm diameter (Fig. 3F, red arrow), indicating that the tumor cells changed their shape to cross through the micro-tunnels (Fig. 3G). The results indicated that the tumor cells could escape from the blood deficient zone to the adjacent, well-perfused host tissues and had the ability to establish new tumor colonies.

**Figure 4:**
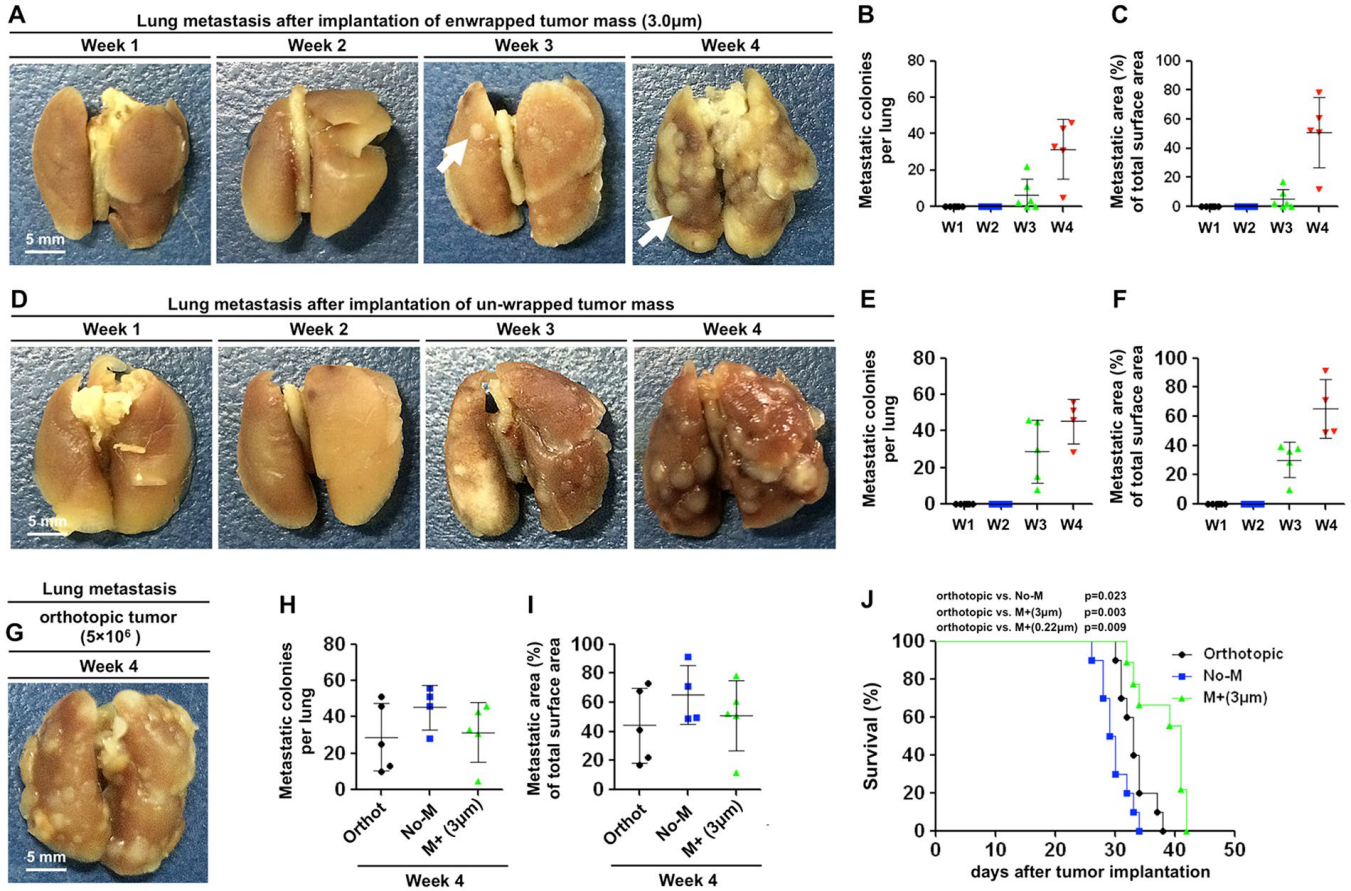
Cutting off tumor blood supply could not prevent the formation of distant metastases. (**A**,**D**) After implantation of a 3.0 μm membrane-wrapped tumor or an un-wrapped tumor for 1, 2, 3, and 4 weeks, the lungs of the tumor-bearing mice were harvested and fixed with 4% paraformaldehyde (PFA) to observe the formation of lung metastases. (**B,E**) The number of tumor colonies on the surface of the lung was counted. (**C,F**) The metastatic lesions were quantified as the percentage of metastatic areas per lung area. The data are presented as scatter plots with means ± SD. The number of dots indicates the “n” of each group. (**G**) Lung metastases from the primary orthotopic breast tumor. (**H,I**) Comparison of the metastatic colonies and metastatic area of the lung surface. Orthot: the orthotopic mammary 4T1 tumor; No-M: the implantation of an un-wrapped tumor mass; M + 3.0 μm: the implantation of a tumor mass wrapped with a 3.0 μm membrane. (**J**) The Kaplan-Meier survival analysis of the mice bearing an orthotopic tumor or implanted tumor (n = 10 per group). The data were analyzed using a log-rank test. The p values are shown within the figure.

More unexpectedly, we found a substantial number of metastatic colonies in the lung, which may ultimately kill the recipient mice (Fig. 4A and Supplementary Fig. S5A). The macro metastatic lesions appeared at week 3, and covered up to 80% of the surface area of the lung at week 4 (Fig. 4A–C). The recipient mice were tumor-free before implantation; thus, the source of the metastases was certainly the tumor that was enclosed by the physical barrier. We then implanted an un-wrapped tumor. In this situation, the vasculature of the host and tumor might be partially re-connected via angiogenesis; thus, in this situation, the blockade of the blood supply was not complete, similar to short-term AA therapy. We found that there were more metastases from the un-wrapped tumor than from the 3.0 μm membrane-wrapped tumors at week 3, although the difference was not significant at week 4 (Fig. 4D–F and Supplementary Fig. S5B,C). We also established an orthotopic breast cancer model by directly inoculating 4T1 cells under the mammary fat pad (Fig. 4G and Supplementary Fig. S5D). Comparison of the metastases at week 4 showed that there were no significant differences between the wrapped tumors and the orthotopic tumors (Fig. 4H,I), indicating that even when the host and tumor vasculature was completely disconnected, development of tumor metastases was not prevented. The Kaplan-Meier survival analysis showed that implantation of the un-wrapped tumors (to mimic short-term AA therapy) led to the worst survival rate (Fig. 4J). The mice implanted with membrane-wrapped tumors (to mimic potent AA therapy) had a slightly increased survival rate compared to mice with orthotopic tumors, but the overall survival was only prolonged for a few days (Fig. 4J). The results are consistent with previous observations that continuous and potent AA therapies provide limited survival benefits, from weeks to months in some cancer patients, whereas short-term AA therapies may worsen the outcome^8^.

### Cutting off blood supply may trigger a cascade response, a “chain reaction”, that may cause uncontrollable tumor dissemination

To determine when the tumor starts to metastasize, one week after implantation, we completely removed the membrane-wrapped tumor. Four weeks later, a substantial number of metastatic colonies were observed in the lung (Supplementary Fig. S6). This suggested that once the blood supply was cut off, the tumor cells started to escape from the blood deficient area, and even when the primary tumor was entirely removed, the escaped cells were enough to establish metastatic colonies.

**Figure 5:**
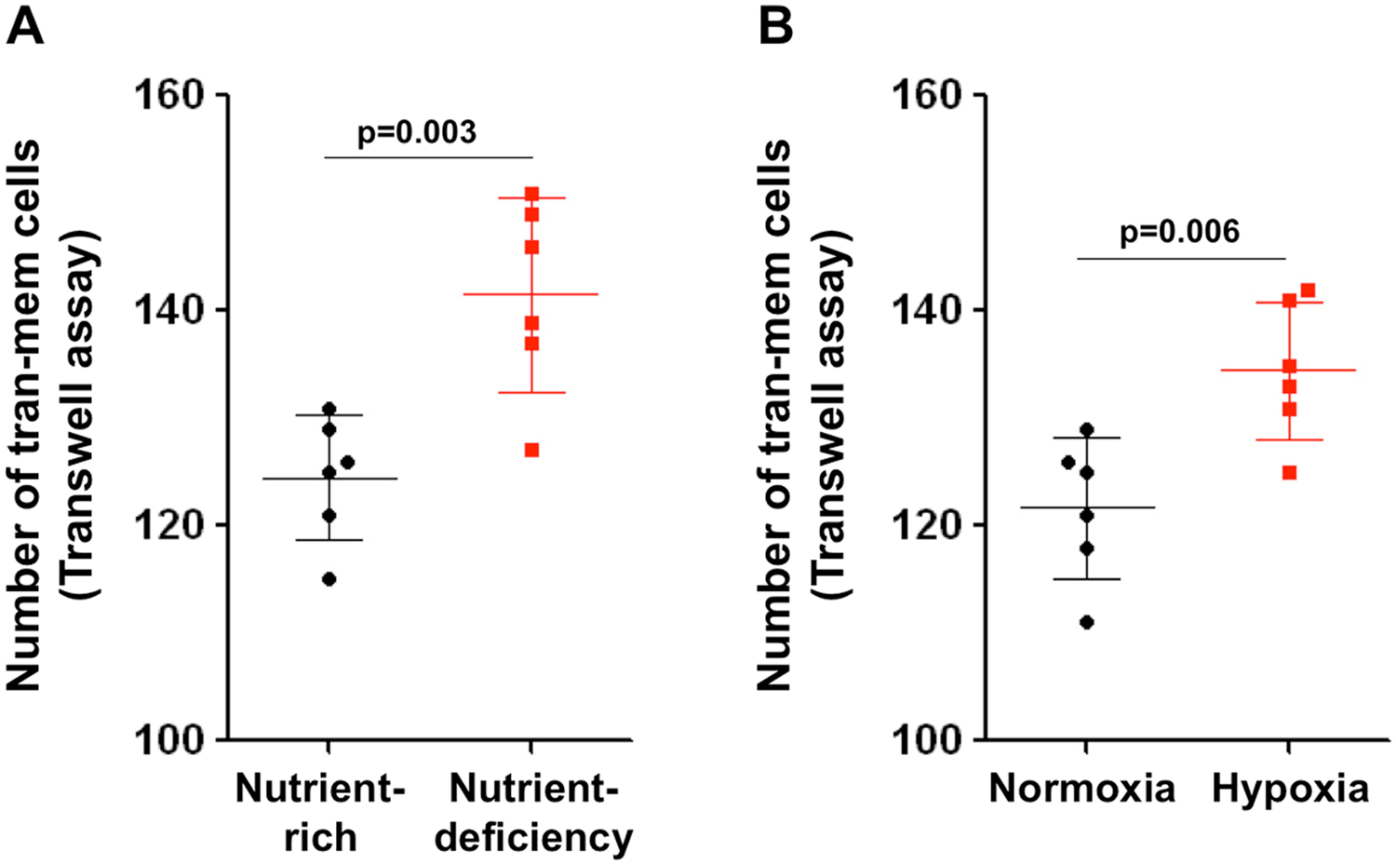
The nutrient-deficiency and hypoxia promoted the invasion ability of tumor cells. (**A**) The tumor cells were seeded on the upper chamber of the transwell system, and cultured in full medium or serum-free medium (to mimic nutrient deficiency). The lower chamber was filled with full medium, making sure that the solution touches the bottom surface of the upper chamber; (**B**) The transwell assay was performed in normoxia or hypoxia (1% O_2_) incubator. The number of cells that invade into the lower chamber was counted after incubation for 24 h (n = 6).

To determine whether or not the environmental stress drives the tumor cells to escape, we performed the transwell assay under nutrient deficiency: The tumor cells were seeded on the upper chamber, and cultured in full medium (DMEM with 10% FBS) or serum-free medium (to mimic nutrient deficiency). The lower chamber was filled with full medium. We want to examine whether the nutrient deficiency will drive the tumor cells migrating to the nutrient-rich area (Fig. 5A). The experiment showed that the cells cultured in the nutrient-deficient chamber had significantly increased migration ability, compared with those in the nutrient-rich chamber. In addition, we performed the transwell assay in normoxia and hypoxia (1% O_2_) incubator. Following incubation for 24 h, we found that the migration ability of the cells in hypoxic environment significantly increased, compared with those in normoxia (Fig. 5B). These results indicated that the environmental stress, including nutrient deficiency and hypoxia, could promote the tumor cells to escape from the starvation area.

**Figure 6:**
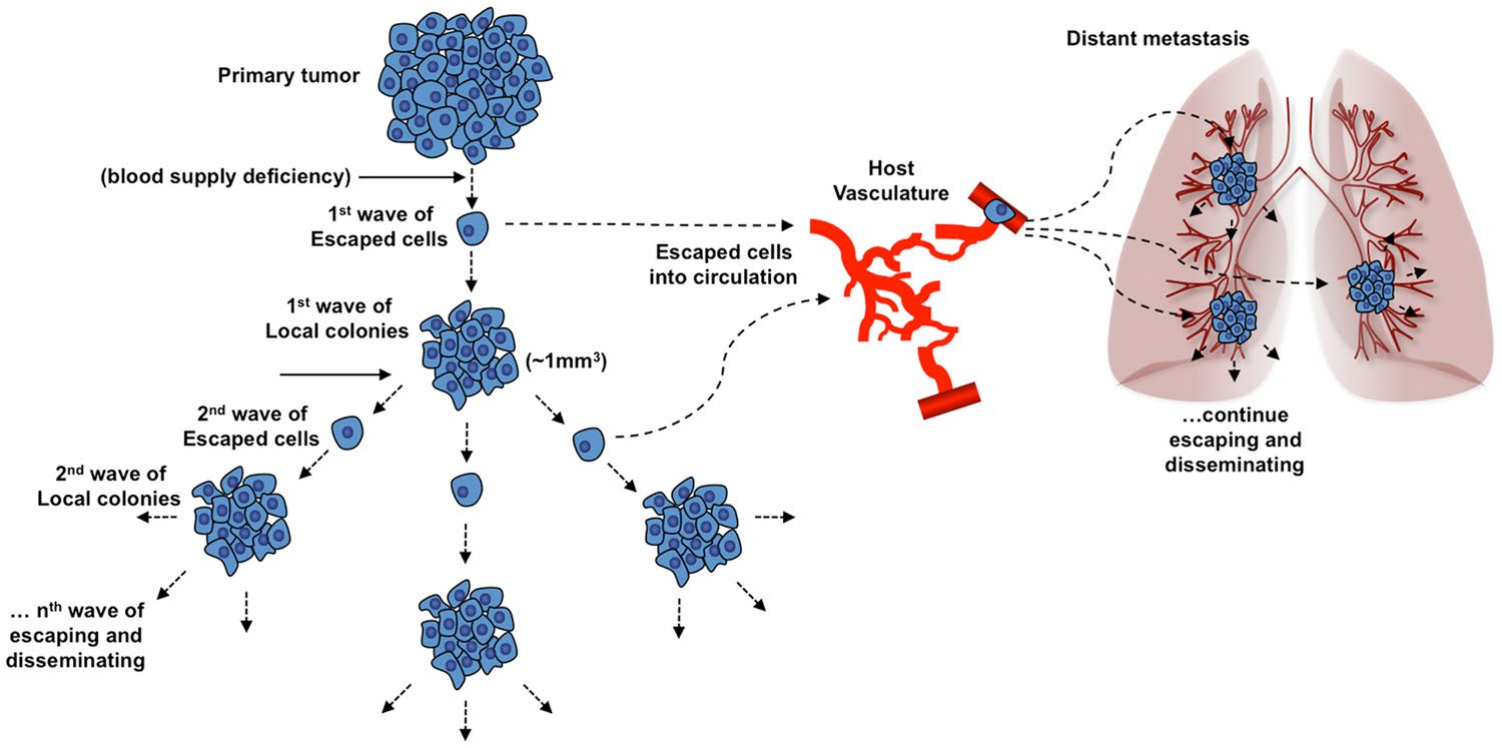
Schematic of the hypothetical tumor dissemination under blood-deficient conditions. Suppose that there is a hypothetical AA agent that can completely cut off the tumor blood supply. It will force the primary tumor cells to escape from the starvation area to the adjacent, well-perfused area and form new clones. When the new tumor colonies reach ~1 mm^3^, the stress of blood deficiency will force the tumor cells to escape and again form new clones. The escaped cells may also have a chance to enter the circulation and colonize in distant organs. If the stress of blood deficiency is sustained, this process would continue as a “chain reaction” that may trigger uncontrollable tumor dissemination.

This made us wonder, if a potent AA agent that could completely block tumor blood supply existed, what would happen? As proposed by the classic tumor-angiogenic theory, when a tumor reaches a volume ~1 mm^3^, it needs neo-vasculature to obtain a blood supply to support its further growth^2, 15^. Suppose that such a hypothetical AA therapeutic destroys the primary tumor and eliminates all of the newly formed tumor colonies by blocking their blood supply when they reach a volume of approximately 1 mm^3^. However, by the very nature of AA therapy itself, it cannot prevent the tumor cells from escaping from the “starvation” zone to the adjacent, well-perfused normal tissues. As the above model demonstrates, the tumor cells can escape from the original zone and establish secondary tumor colonies at other locations. When these new colonies grow and reach ~1 mm^3^, they are supposed to be killed again by the hypothetical AA therapeutic, however, the second wave of escaped cells will establish the next level of tumor colonies. If this process continues, it may trigger a cascade response, a “chain reaction”, that may lead to uncontrollable tumor dissemination (Fig. 6).

We used a mathematical method to simulate this process: let ***x***_***0***_ be the initial number of the primary tumor, and let ***x***_***1***_ be the number of tumor colonies formed by the first wave of escaped cells and ***x***_***n***_ be the number of colonies after ***n*** rounds of escape, where ***n*** = 1, 2, 3…. Let ***T*** (days) be the time for the escaped cells to form a new colony with a volume of ~1 mm^3^, and let ***N*** be the number of escaped cells from one colony in ***T*** days and ***M*** be the minimal number of cells required to establish a new tumor colony. After ***t*** days, the tumor cells will escape $n=[\frac{t}{T}]$ rounds, where $[\frac{t}{T}]$ is the integer part of $\frac{t}{T}$. Thus, we have the following model: the number of colonies at time ***t*** will be $x(t)={(\frac{N}{M})}^{[\frac{t}{T}]}{x}_{0},t\ge T.$

Note that a drug that can completely block the tumor blood supply does not exist. In addition, even tumor colonies of less than 1 mm^3^ spread throughout the body, and it is difficult to detect them all using current technology. Thus, this model should not be considered a precise calculation formula, but a fuzzy prediction tool to predict whether an anti-angiogenic strategy will result in tumor dissemination. According to this model, the greater the ability of the tumor cells to escape (the higher the value of ***N***) and form new tumor clones (the lower the value of ***M***), the faster they spread in the body. Given that a tumor has high metastatic potential $(\frac{N}{M} > 1)$, the number of tumor colonies will increase over time. On the other hand, for tumors with low metastatic potential $(\frac{N}{M}\le 1)$, the number of tumor colonies ***x(t)*** after ***t*** days will be less than the initial number ***x***_***0***_. In other words, for low metastatic tumors, AA therapy will not cause tumor dissemination, and these tumors may benefit from AA cancer therapy.

**Figure 7:**
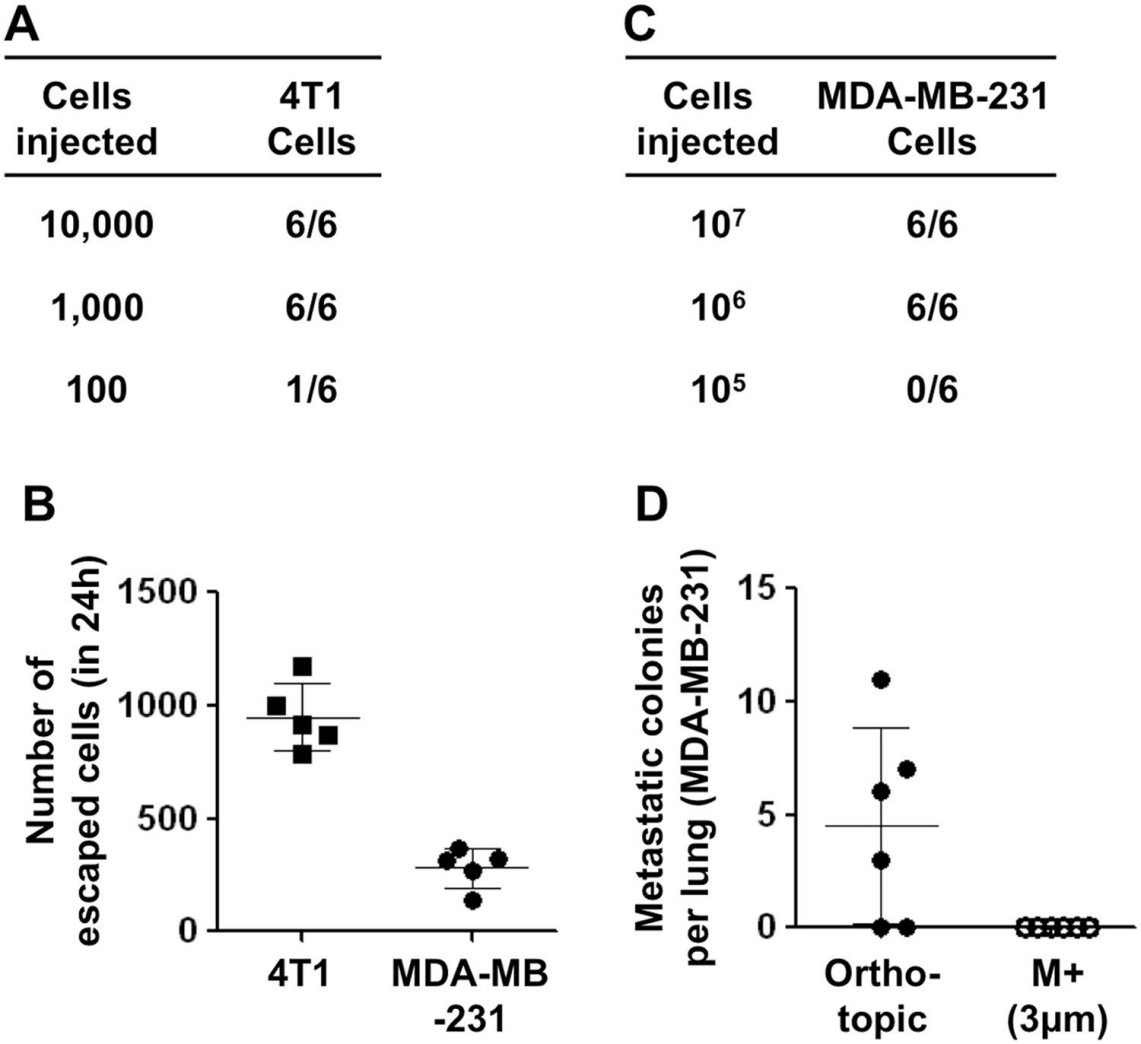
Validate the fuzzy prediction model using the experimental data. (**A,C**) Tumorigenic ability assay using 4T1 and MDA-MB-231 cells (n = 6). (**B**) Count the number of tumor cells that escaped from the membrane-wrapped tumor in 24 h (n = 5). (**D**) Comparison of lung metastatic colonies from the orthotopic and membrane-wrapped MDA-MB-231 tumor (n = 6). The data are presented as scatter plots with means ± SD.

We next validated this fuzzy prediction model using the experimental data. The tumorigenic ability assay showed that 1000 was the lowest number of 4T1 cells required to establish an orthotopic tumor under the mammary fat pad, and it took 7 days for the tumor to grow to a volume of approximately 1 mm^3^ (Fig. 7A). Therefore, the value of ***M*** and ***T*** was estimated to be 1000 and 7. We then counted the number of cells that escaped from the 3.0 μm membrane-wrapped 4T1 tumor (with a diameter of 5 mm). This experiment showed that approximately 950 4T1 cells could cross through the membrane in 24 h (Fig. 7B). Therefore, the ***N*** value, the number of cells escaped from the wrapped tumor in 7d, was estimated to be 950 × 7 = 6650. Because ***N*** = 6650, ***M*** = 1000, and $\frac{N}{M} > 1$, according to the formula $x(t)={(\frac{N}{M})}^{[\frac{t}{T}]}{x}_{0},t\ge T$, the number of tumor colonies will increase exponentially over time. As presented above, after the membrane-wrapped 4T1 tumor was implanted for 4 weeks, we observed substantial locally invasive and distant metastatic colonies spreading widely throughout the body. Thus, the experimental data fit the prediction of our model.

We next used MDA-MB-231 cells, a metastatic human breast cancer cell line, to validate this model. The tumorigenic ability assay showed that the lowest number of MDA-MB-231 cells needed to establish an orthotopic tumor was 10^6^ (Fig. 7B), and it took 10 days for the tumor to grow to ~1 mm^3^. The trans-membrane cell count showed that 311 cells could cross through the membrane in 24 h (Fig. 7C). Thus, the ***N*** value of the MDA-MB-231 tumor was estimated to be 311 × 10 = 3110, the ***M*** value was 10^6^, and the ***T*** value was 10. Because $N\ll M$, according to the formula $x(t)={(\frac{N}{M})}^{[\frac{t}{T}]}{x}_{0},t\ge T$, the number of tumor colonies will never exceed its initial number, ***x***_***0***_. In other words, the model predicts that AA therapy will not cause the MDA-MB-231 tumor to disseminate. In fact, after implantation of the membrane-wrapped MDA-MB-231 tumor for 4 weeks, no local invasion or distant metastatic tumor colonies were found outside the physical barrier (Fig. 7D). Thus, the experimental data fit the prediction of our model, which indicated that potent AA therapy would not induce a low metastatic tumor to invade or metastasize. The above results suggest that the anti-angiogenic strategy may only be of benefit if the invasion and clone formation ability of a cancer is relatively weak and meets the condition $\frac{N}{M}\le 1$.

### The tumor malignancy could be increased during the tumor escaping process

**Figure 8:**
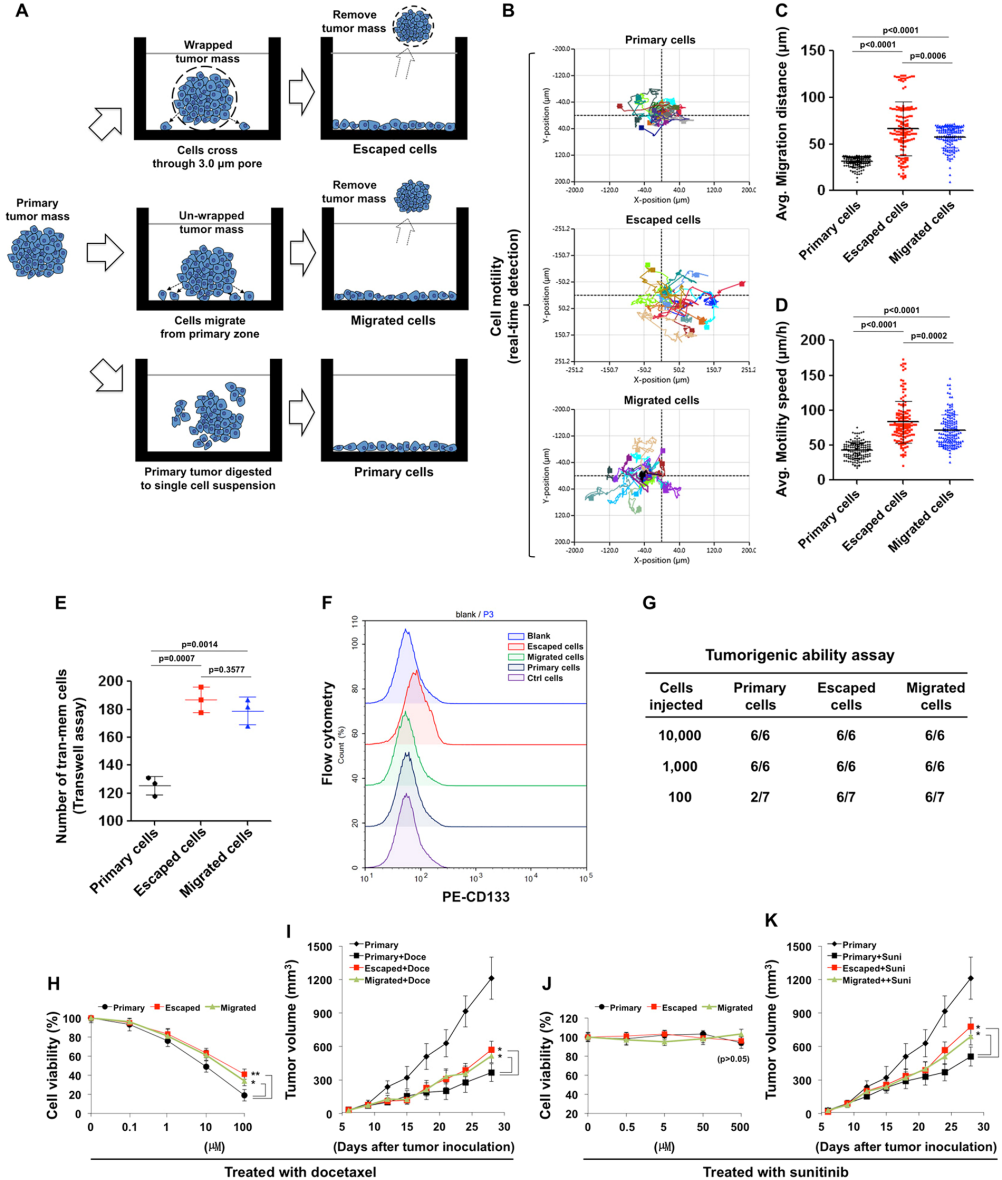
The tumor malignancy was further increased during the tumor escaping process. (**A**) The encapsulated 4T1 tumor was incubated in the 6-well plate. After the tumor cells escaped through the micro-pores, the tumor mass was entirely removed, and the cell remaining on the bottom of the plate were named as “Escaped cells”. The cells migrated from the un-wrapped tumors were named as “Migrated cells”. The primary orthotopic tumor was directly minced and digested to single cell suspension, and named as “Primary cells”. (**B–D**) The cells were then subjected to the real-time detection of cell motility. 19 cells of each group were randomly chosen for the analysis. (**B**) The motion trail of each chosen cell. (**C**) The average migration distance (μm) and (**D**) the average motility speed (μm/h) of the 19 cells at each time point was calculated using the software HoloMonitor ver.2.6.1. The cells were also subjected to transwell assay to evaluate the invasion ability (**E**) and flow cytometry to detect CD133 (**F**). (**G**) The tumorigenic ability of the cells was determined by limiting dilution assay. (**H,J**) The cell viability was measured after the treatment of docetaxel or sunitinib. (**I,K**) 2 × 10^6^ Primary, Escaped, or Migrated cells were injected into the mammary fat pad. After 6 days of inoculation, mice were treated with docetaxel (5 mg/kg) or sunitinib (40 mg/kg), and the tumor volume was measured every 3 day (n = 6). Statistical differences between groups were examined using the 2-tailed Student’s *t-*test, and a p value < 0.05 was considered statistically significant (*p < 0.05; **p < 0.01).

The secondary tumors, which were forced to escape from the original tumor zone, might acquire some properties that could further increase its invasive ability, and might become resistant to the follow-up anti-angiogenic therapy and chemotherapy. To determine whether the tumor malignancy is affected or not, we collected the cells escaped from the enwrapped tumor, the cells migrated from the surface of the un-wrapped tumor, and the primary tumor cells themselves, as indicated in Fig. 8A. The cells were named Escaped cells, Migrated cells, and Primary cells, respectively. The real-time detection of cell motility showed that the motion abilities of the Escaped cells and Migrated cells were much higher than that of the Primary cells (Fig. 8B–D). The transwell assay showed similar results (Fig. 8E). The flow cytometry showed that the Escaped cells increased the expression of CD133, indicating that they may acquire some stem-like properties (Fig. 8F). The 4T1 cancer is highly malignant in itself, and 1,000~10,000 cells are enough to form tumor *in situ*. We found that the Escaped cells and Migrated cells were even more malignant, with 100~1000 cells enough to form *in situ* tumor (Fig. 8G).

In addition, we used sunitinib and docetaxel to examine whether or not the sensitivity of the secondary tumor cells to chemo- or AA therapy were altered. The cytotoxicity assay showed that the Escaped and Migrated cells were less sensitive to the treatment of docetaxel *in vitro*. However, sunitinib showed not direct killing activity on the cultured tumor cells (Fig. 8H,J). An *in vivo* study was also performed: the mice were injected with 2 × 10^6^ Primary (original 4T1 cells), Escaped, and Migrated cells, respectively. After 6 days of tumor inoculation, the mice were received the treatment of docetaxel or sunitinib. The therapeutic experiments showed that the sensitivity of the secondary tumor to chemo- or anti-angiogenic therapy was significantly decreased, compared to the original 4T1 tumor (Fig. 8I,K). However, the mRNA level of GLUT1, which substantially increases glucose import into the cytoplasm and may be responsible for the “Warburg effect” of cancer cells^1^, did not change in those cells (Supplementary Fig. S7). It suggested that the cell metabolic characteristics associated with GLUT1, at least in this condition, might not change.

Taken together, the above results suggested that the malignancy of tumor cells could be further increased during the escaping and migrating process. It was like a kind of “self-reinforcement”, which might in turn enhance the tumor cells to escape and metastasize again.

## Discussion

To date, more than 40 molecules have been determined to play a role in angiogenesis^4, 11^. Unless we can target them all, it is nearly impossible to completely block tumor angiogenesis. Here, we designed a model to circumvent this obstacle by disconnecting the vasculature between the tumor and the host using a physical barrier. The micro-pores on the barrier allowed material exchange at the tumor/host border, but the neo-vessels generated by angiogenesis could not cross through the barrier to connect the host and tumor vasculature. Here, we did not use impenetrable materials to enclose the tumor because it would be equivalent to completely isolating the tumor from the host, which is no different than a surgical resection. This model may be simple, but it created an environment in which the blood flow from the host to the tumor was completely cut off. Meanwhile, the host vasculature was not affected, and both of these conditions fit the characteristics of an ideal AA therapy. With the aid of this model, we were able to learn about what would occur if the tumor blood supply was completely cut off.

To our surprise, we found that even when the tumor blood supply was completely blocked, it neither eliminated the primary tumor cells nor prevented local invasion or formation of distant metastases. The result seems contrary to our expectation for an ideal AA therapy. Be that as it may, if we look into the anti-angiogenic strategy deeply, we may find that this result is not so unexpected. An AA drug is designed to inhibit tumor angiogenesis. According to the theoretical basis of this strategy, AA therapy does not prevent cancer cells from migrating through the tissue interspace via their own motility. In addition, tumor cells may migrate along the preexisting vessels of the host tissues via “vessel co-option” (tumor cells hijacking the existing host vasculature or migrating along the vessels of the host organ^16, 17^), which may be an important way for tumor cells to escape from the starvation area. Thus, AA therapy creates an extreme environment of nutrient and oxygen deficiency, while it cannot prevent cancer cell migration, which may lead to an inevitable consequence: the tumor cells will be forced to escape from the “starvation” area to other locations. There is no doubt that the primary tumor will shrink because of blood deficiency. However, if the cancer cells are driven away from the blood deficient area, they will escape continuously under the consistent environmental stress until they spread throughout the body. Thus, even if the tumor invasiveness remains unchanged, the “blood deficiency” itself will be sufficient to cause the tumor to metastasize. In most cases, metastatic lesions, not primary tumor growth, kills cancer patients^18, 19^. Therefore, what benefits can we get from AA therapy?

As is well known, the classic tumor angiogenic theory has a basic assumption: tumor cells can give rise to dormant, microscopic tumors of ~1 mm^3^ in the absence of angiogenesis, but these micro tumors are harmless to the host^2, 15, 20, 21^. However, the ability to invade and metastasize may be one of the most important hallmarks of cancer^1^, which makes it unlikely that cancer cells, especially highly metastatic ones, would go dormant simple because they lack a blood supply. This may be a theoretical flaw of the classic anti-angiogenic theory, which overlooks the inherent ability of cancer cells to migrate, invade, and colonize in new locations. As presented above, highly metastatic cancer cells, such as 4T1 cells, can escape from the “starvation” area and form new colonies in the adjacent tissues and distant organs. Envision that a potent AA therapy exists that can create a blood deficient environment surrounding each tumor colony, similar to the model that we established above. By using a mathematical method to simulate this process, we obtained the model $x(t)={(\frac{N}{M})}^{[\frac{t}{T}]}{x}_{0},t\ge T$, which indicated that potent AA therapy would cause some tumors, particularly the highly metastatic ones where $(\frac{N}{M} > 1)$, to spread exponentially. On the other hand, for tumors with low metastatic potential, where $(\frac{N}{M}\le 1)$, the number of tumor colonies will not exceed the initial number ***x***_***0***_. We can say that only under this condition will an anti-angiogenic strategy benefit cancer patients.

Several reports have shown that some potent AA therapies could promote tumor metastasis^7, 8^. Herein, we were not were not trying to challenge or re-invent this concept, but sought to analyze the phenomenon that AA therapy may trigger tumor dissemination in an alternative way. We created a complete blood-deficient environment, observed the behavior of tumor cells in it, and established a mathematical model to simulate this process. Our data suggest that the increase tumor metastases may not be an isolated phenomenon but, instead, may be an inevitable consequence of the anti-angiogenic strategy. AA therapy will create an extreme environment of hypoxia and nutrient stress, which may lead to an inevitable consequence: the cancer cells that can tolerate the blood deficiency are “naturally selected” and survive, whereas the consistent environmental stress promotes a portion of cells to escape from the “starvation” area to another, more well-perfused, area. This may be the “inherent vice” of the anti-angiogenic strategy. Even the possibility that higher metastatic phenotype may be induced is not a consideration, the blood deficiency itself, will be sufficient to cause some tumors, particularly the highly metastatic ones, to spread more aggressively. According to our simulation, the anti-angiogenic strategy may only be of benefit if the cancer metastatic potential is relatively low, and meets the condition $\frac{N}{M}\le 1$; however, in the majority of clinical human cancers, which usually have a high metastatic potential, $(\frac{N}{M} > 1)$, AA therapy will inevitably promote cancer dissemination and formation of metastases.

Herein, we used two spontaneously metastatic tumors, MDA-MB-231 and 4T1 to validate this prediction model. Not all tumor cells can migrate through 3.0 μm pores. The stiffness of the nucleus, which is mainly regulated by the ratio of Lamin A/B, is important for tumor cells to penetrate through tight interstitial spaces. Dicher^22, 23^ and Lammerding^24–26^ labs did significant works in this field. However, although both 4T1 and MDA-MB-231 could cross through the 3.0 μm pores, as shown in Fig. 7, only 4T1 produced local invasive and distant metastatic lesions outside the barrier. Thus, the ability to cross through 3.0 μm pores might not be the major cause of the different invasive ability between the two cells. We found that although MDA-MB-231 cells had the ability to across the 3.0 μm pores, its tumorigenic ability was relatively weak, resulting in a high *M* value (10^6^). According to the formula $x(t)={(\frac{N}{M})}^{[\frac{t}{T}]}{x}_{0},t\ge T$, because $N\ll M$, the number of tumor colonies will never exceed its initial number, *x*_*0*_. In other words, cutting off blood supply will not cause the MDA-MB-231 tumor to disseminate. Thus, the ratio of *N/M*, that is the escaping ability vs. tumorigenic ability, may determine whether AA therapy will cause metastasis or not.

For a long time, people have been looking for AA drugs that are more potent than the existing ones. Ironically, our study suggests that even if we could develop a “perfect” AA therapy that completely inhibited tumor angiogenesis without affecting normal blood vessels, it might, instead, cause the cancer, particularly highly metastatic ones, to spread more aggressively. In addition, although the AA drugs that are currently used can prolong the life of certain cancer patients for weeks to months, the longer the patients receive AA therapy, the longer the tumors are under environmental stress, such as hypoxia and nutrient deficiency. AA therapies may prolong life for a short time, but in the long run, the escaped cells and the secondary tumors formed by them, may become more malignant and resistant to follow-up therapies (as shown in Fig. 8), which may cause the tumor to be more difficult to be eliminated. The benefit and potential harm of the anti-angiogenic strategy may need to be reconsidered. If we are looking for a cure or at least long-term remission for cancer patients, anti-angiogenic strategy may not be our best answer.

## Experimental Procedures

### Cells, Antibodies and Reagents

4T1 mouse mammary cancer cells · were obtained from American Type Culture Collection (ATCC) and routinely cultured according to ATCC guidelines. Antibody for CD31 (Cat. 550274) was from BD Biosciences. Antibody for Ki-67 (Cat. RM-9106) and the AlexaFluor488 or 568 conjugated secondary antibodies were from Thermo Fisher Scientific. The FITC-conjugated dextran (Cat. FD2000S) was from Sigma Aldrich.

### Tumor Model

All animal experiments were approved by the Animal Ethics Committee of Sichuan University and performed according to institutional and international guidelines. Balb/c mice at 6–8 weeks of age were injected subcutaneously with 2.5 × 10^6^ 4T1 cells into the right flank. When the tumor reached a diameter of approximately 5 mm, it was surgically removed and enclosed in a hydrophilic polycarbonate membrane with 3.0 μm pore size (from Millipore, Cat. TSTP04700). The membrane-wrapped tumor was tied using surgical sutures, and the opening was heat-sealed. The encapsulated tumor was then implanted into a healthy anaesthetized mouse. The operative procedure is shown in Fig. 1A.

### Analysis of Blood Perfusion of the Host and Tumor Vasculature

Three weeks after implantation, FITC-dextran (4 mg/kg in 100 μl PBS) was injected into the anaesthetized tumor-bearing mice through the left ventricle. After 10 min, the membrane-wrapped tumor and the adjacent tissues were carefully dissected and immersed in liquid nitrogen. The frozen samples were embedded in the OCT compound (Leica), cut into 10~20 μm thick cryostat sections, mounted on gelatin/potassium dichromate coated slides, and stored at −80 °C until needed. Before use, the cryo-slides were warmed to room temperature (RT) for 30 min and fixed in ice-cold acetone for 25 min. The slides were stained with rat-anti-mouse CD31 (1:50) followed by AlexaFluor 568-conjugated goat-anti-rat IgG (1:200) as a marker of total vasculature. Co-localization of red and green fluorescence was considered to represent blood-perfused vessels.

### Analysis of the Implanted Tumor within the Physical Barrier

The enclosed tumor with its adjacent tissues was collected after implantation for 1, 2, 3, and 4 weeks. The samples were stained with H&E for general observation. The necrotic area (%, including the coagulative and liquefactive necrosis) of 10 samples from each group is presented as scatter plots with mean ± S.D. The number of the cells with an intact nucleus per non-necrotic field was counted manually in 20 fields and presented as scatter plots with mean ± S.D. The slides were also stained with Ki67 (1:100) to show the proliferating tumor cells and stained with DAPI (1 μg/ml) to display the cell nucleus. The number of Ki67 and DAPI double-positive cells per DAPI-positive area was counted manually in 20 fields. Note that the Ki67^+^ signals that were not co-localized with DAPI were cell debris and were excluded from counting.

### Analysis of the Local Invasive and Distant Metastatic Tumors

After the implantation of the tumors for 1, 2, 3, and 4 weeks, the tumor samples and lungs were collected and fixed with 4% PFA for 2 h. The tumor volume (mm^3^) was calculated using the formula: 1/2 × length × width × width. The newly formed tumor volume equals the total tumor volume minus the primary tumor volume. The number of surface metastatic lesions in the lungs was counted manually. The metastatic area was expressed as the percentage of lung surface covered with metastatic lesions. The data is presented as scatter plots with mean ± S.D.

### Count the Number of Tumor Cells that Escaped from the Encapsulated Tumor

When the subcutaneous tumor reached a diameter about 5 mm, it was surgically removed, enwrapped with the 3.0 μm membrane, and placed into the 6-well plates that were pre-coated with 0.7% agarose as the bottom layer. The membrane-enwrapped tumors were immersed and incubated in the 0.7% agarose with DMEM plus 10% FBS. After 24 h, the membrane-enwrapped tumors were completely removed, and the agarose medium was transformed into liquid medium by heating. The cells that escaped into the medium were collected by centrifugation and re-suspended in PBS, and the cell number was counted under microscope.

### Isolation of the Escaped cells, Migrated cells, and Primary cells

The membrane-wrapped 4T1 tumor was placed in the 6-well plate and cultured in the complete DMEM with 10% FBS. When the cells escaped through the membrane and attached the bottom of plate, the enwrapped tumor mass was entirely removed, and the attached cells was named “Escaped cells” for later use. Similar to the above, the un-wrapped tumor was cultured in the 6-well plate until the cells migrated from the tumor surface to the bottom of plate. The tumor mass was removed, and the attached cells were named “Migrated cells”. The orthotopic tumor mass was surgically removed, minced through a 75 μm nylon mesh, and digested with type I and type IV collagenases to isolate the Primary cells.

### Real-time Cell Motility Detection

The Escaped cells, Migrated cells, and Primary cells were routinely cultured in the 24-well plate until the cells grew to semi-confluent. The cells were imaged with the HoloMonitor M4 (Phase, Sweden) during 10 hours, 4 minutes between image frames. 19 cells of each group were randomly chosen for the analysis. The motion trail of each cell was generated automatically. The average migration distance (μm) and the average motility speed (μm/h) at each time point were calculated using the software HoloMonitor ver.2.6.1, and presented as scatter plots with means ± SD.

### Transwell Assay

5,000 4T1 cells were seeded into the upper chamber (transwell inserts, pore size: 3.0 μm, Millipore) of the 24-well plates and subsequently incubated at 37 °C, 5% CO2. Following incubation for 24 h, transmigrated cells on the lower surface of the membrane were stained with CFSE, fixed with 4% PFA. The total number of migrated cells was counted under microscope. The experiment was performed in triplicate and repeated three times independently.

### Image Analysis and Statistics

Immunostained sections were examined using a Zeiss Z2 fluorescence microscope. The images were analyzed using Image-Pro Plus analysis software (version 5.0.9.2). Statistical differences among groups were examined using the 2-tailed Student’s *t-*test, and a p value < 0.05 was considered statistically significant. The survival analysis was performed using Kaplan-Meier curves and the log-rank test. The curves and p values were calculated using the software GraphPad Prism 5.0, and a p value < 0.05 was considered statistically significant.

## Electronic supplementary material

SUPPLEMENTARY INFO
