# Supplementary material for: The “inherent vice” in the anti-angiogenic theory may cause the highly metastatic cancer to spread more aggressively
Source: Sci Rep. 2017 May 24;7:2365. doi: 10.1038/s41598-017-02534-1 (PMC5443774; doi:10.1038/s41598-017-02534-1)
Supplement: Supplementary file 1 — SUPPLEMENTARY INFO [file 41598_2017_2534_MOESM1_ESM.pdf]

(Supplementary figures)

The "inherent vice" in the anti-angiogenic theory may cause the highly metastatic cancer to spread more aggressively

Denian Wang M.S.[1,2], **Chun Tan** M.S.[1,2], **Fei Xiao** M.D.[3], **Lan Zou** Ph.D[4]., **Lijun Wang** Ph.D.[2], **Yong'gang Wei** M.D.[5], **Hanshuo Yang** Ph.D.[6,7], **Wei Zhang** Ph.D.[2,7]

1 The authors contributed equally to this work

2 Molecular Medicine Research Center, West China Hospital, State Key Laboratory of Biotherapy/Collaborative Innovation Center of Biotherapy, Sichuan University, Chengdu 610041, Sichuan, P. R. China

3 Department of Intensive Care Unit of Gynecology and Obstetrics, West China Second University Hospital, Sichuan University, Chengdu, 610041, Sichuan, P. R. China

4 Department of Mathematics, Sichuan University, Chengdu, Sichuan 610064, P. R. China.

5 Department of Liver Surgery, West China Hospital, Sichuan University, Chengdu, 610041, Sichuan, P. R. China

6 State Key Laboratory of Biotherapy and Cancer Center, West China Hospital, and Collaborative Innovation Center of Biotherapy, Chengdu, Sichuan 610064, P. R. China.

7 Correspondence should be addressed to: Wei Zhang: zhangwei197610@163.com (or Hanshuo Yang: yhansh@scu.edu.cn)

Address: No. 1, Ke Yuan 4th Road, Gao Peng Street, Chengdu, Sichuan, 610041, P. R. China

Supplementary Figures

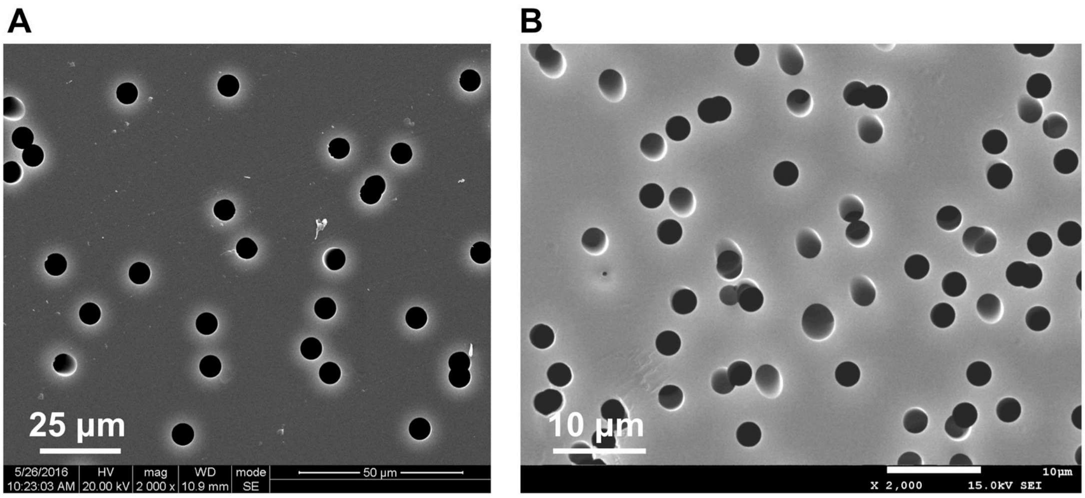

TETP04700 | hydrophilic membrane with 8.0 µm pore size
(Electron microscopic image)

TSTP04700 | hydrophilic membrane with 3.0 µm pore size
(Electron microscopic image)

Supplemental Figure S1. The electron microscopy scanned the surface of the membrane with 8.0 µm (**A**) and 3.0 µm (**B**) micro-pores.

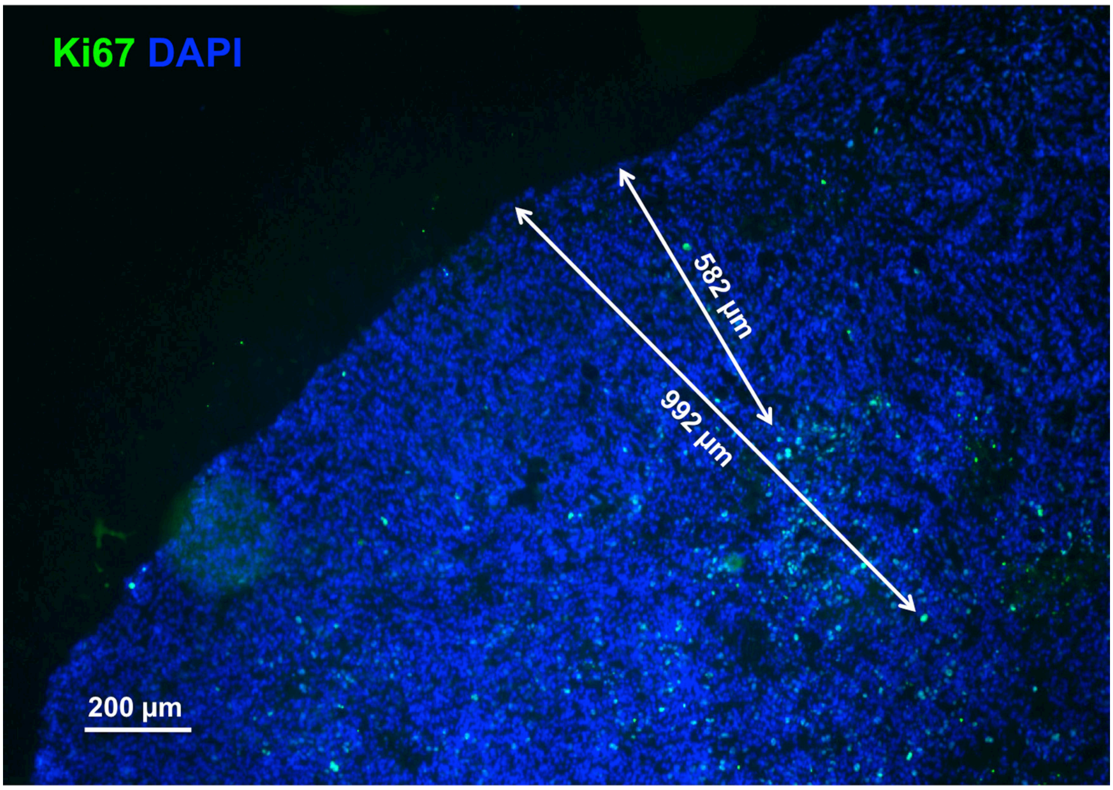

Supplemental Figure S2. Some Ki67^{+} cells resided deeply into the core of the encapsulated tumor.

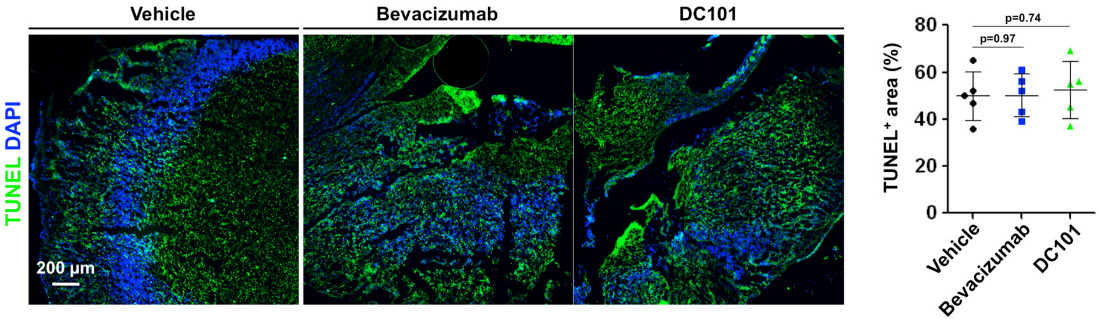

Supplemental Figure S3. The encapsulated tumors treated with vehicle, bevacizumab, DC101 were subjected to TUNEL assay.

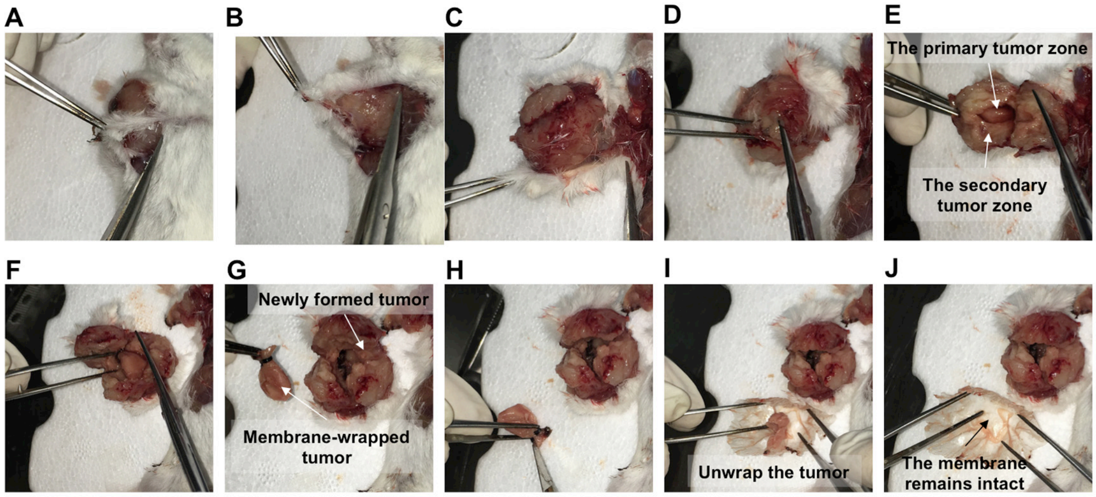

Supplemental Figure S4. Isolation of the enwrapped tumor. (A-D) After tumor implantation for 4 weeks, the mice were anesthetized, and the implanted tumor with the adjacent tumor tissues were entirely isolated by blunt dissection. (**E**) Cut into the tumor, display the enwrapped tumor mass that was enclosed by the secondary tumor tissues. (**F,G**) Isolate the enwrapped tumor from the newly formed tumor tissues. (**H,I**) Cut off the heat-sealing line, and untangle the suture that sealed the membrane. (**J**) The membrane is unbroken and remains intact.

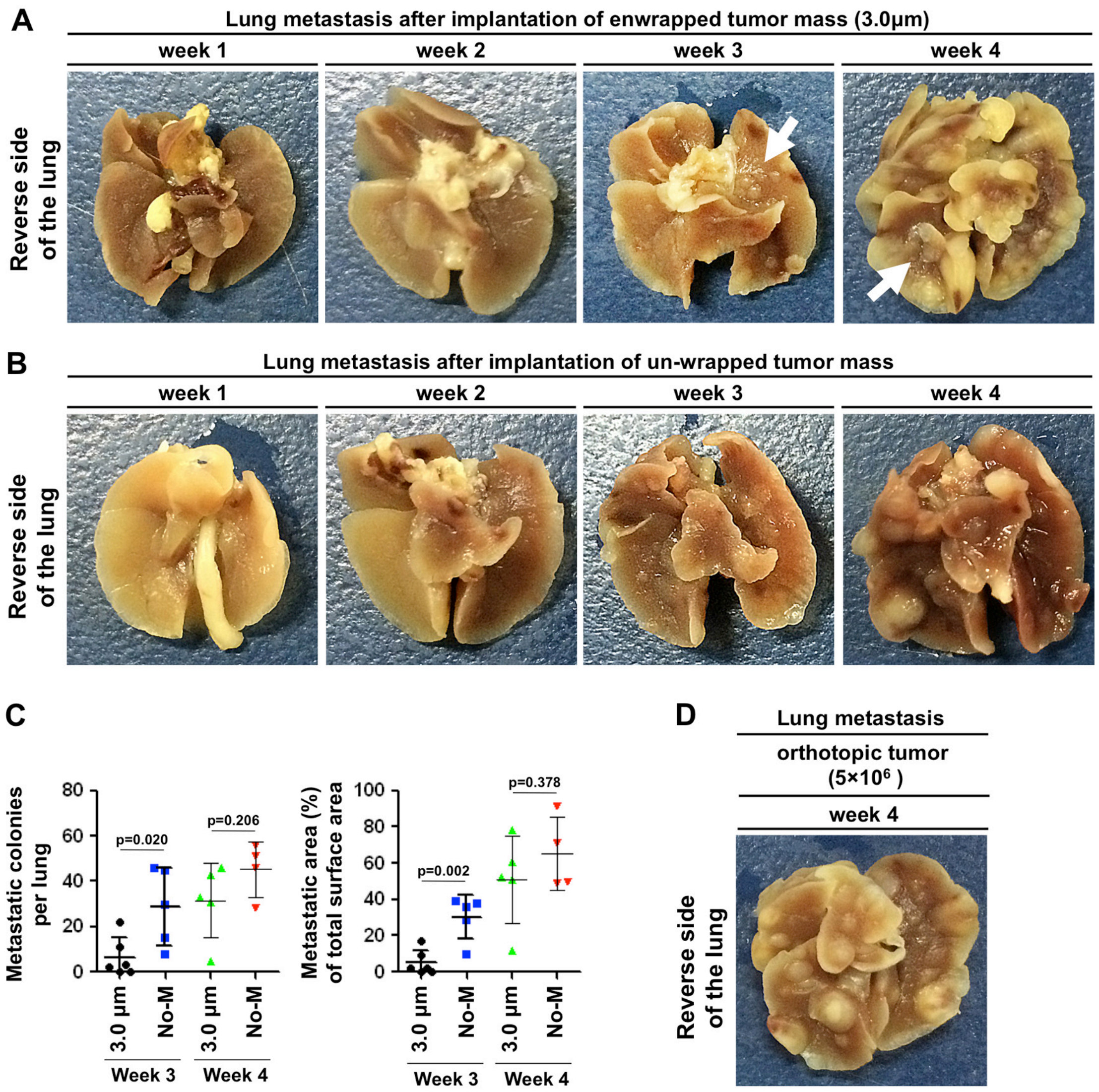

Supplemental Figure S5. Cutting off tumor blood supply couldn't prevent the distant metastasis. (**A,B**) After the implantation of the membrane-wrapped tumor or the un-wrapped tumor for 1, 2, 3, and 4 weeks, the lungs of the tumor-bearing mice were harvested and fixed with 4% paraformaldehyde (PFA) for the observation of lung metastasis. The representative images showed the reverse side of the lungs. (**C**) The number of tumor colonies and the relative metastatic area on the surface of lung in the 3.0 µm membrane-wrapped tumor and un-wrapped tumor at week 3 and week 4. The Data are presented as scatter plots with means ± SD. The "p" values were shown within the image. (**D**) The representative images showed the reverse side of the lungs metastasis from the orthotopic breast tumor.

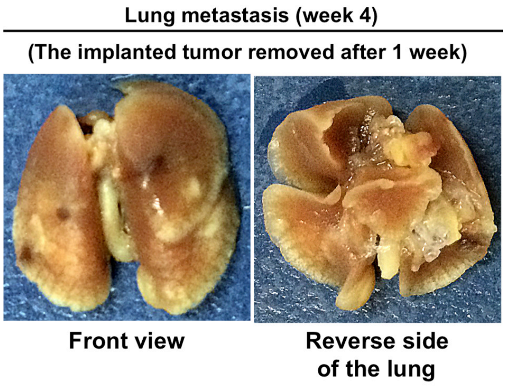

Supplemental Figure S6. Even if the primary tumor was completely removed, the escaped cells were enough to establish metastatic colonies. The membrane-wrapped tumor was completely removed after implantation for 1 week. After 4 weeks, the mice were sacrificed and the lungs were isolated. The representative images showed the metastatic colonies on the surface of the lung.

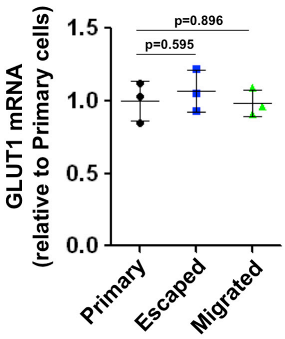

Supplemental Figure S7. GLUT1 expression was not change. The Primary, Escaped, and Migrated cells were subjected to Real-time PCR to detect the mRNA level of GLUT1.
